# Macro level factors influencing strategic responses to emergent pandemics: A scoping review

**DOI:** 10.7189/jogh.11.05012

**Published:** 2021-07-01

**Authors:** Nina J Zhu, Ewan B Ferlie, Enrique Castro-Sánchez, Gabriel Birgand, Alison H Holmes, Rifat A Atun, Hailey Kieltyka, Raheelah Ahmad, the COMPASS (COntrol and Management of PAndemicS through Strategic analysis) study group

**Affiliations:** 1 National Institute for Health Research Health Protection Research Unit in Healthcare Associated Infection and Antimicrobial Resistance at Imperial College, London, UK; 2 King’s Business School, King’s College London, London, UK; 3 Division of Nursing, School of Health Sciences, City, University of London, London, UK; 4 Department of Global Health and Population, Harvard T. H. Chan School of Public Health, Boston, MA, USA; 5 Division of Health Services Research and Management, School of Health Sciences, University of London, London, UK; 6 Institute of Business & Health Management, Dow University of health Sciences, Karachi, Pakistan

## Abstract

**Background:**

Strategic planning is critical for successful pandemic management. This study aimed to identify and review the scope and analytic depth of situation analyses conducted to understand their utility, and capture the documented macro-level factors impacting pandemic management.

**Methods:**

To synthesise this disparate body of literature, we adopted a two-step search and review process. A systematic search of the literature was conducted to identify all studies since 2000, that have 1) employed a situation analysis; and 2) examined contextual factors influencing pandemic management. The included studies are analysed using a seven-domain systems approach from the discipline of strategic management.

**Results:**

Nineteen studies were included in the final review ranging from single country (6) to regional, multi-country studies (13). Fourteen studies had a single disease focus, with 5 studies evaluating responses to one or more of COVID-19, Severe Acute Respiratory Syndrome (SARS), Middle East Respiratory Syndrome (MERS), Influenza A (H1N1), Ebola virus disease, and Zika virus disease pandemics. Six studies examined a single domain from political, economic, sociological, technological, ecological or wider industry (PESTELI), 5 studies examined two to four domains, and 8 studies examined five or more domains. Methods employed were predominantly literature reviews. The recommendations focus predominantly on addressing inhibitors in the sociological and technological domains with few recommendations articulated in the political domain. Overall, the legislative domain is least represented.

**Conclusions:**

Ex-post analysis using the seven-domain strategic management framework provides further opportunities for a planned systematic response to pandemics which remains critical as the current COVID-19 pandemic evolves.

The current SARS-CoV-2 (COVID-19) pandemic has brought into sharp focus the readiness and capacities of health and wider systems in the ability to respond and protect the public [1,2]. Real-time situational analyses [3] are essential as the pandemic evolves, but this learning must build on what is already known from (albeit smaller scale) pandemics, and the role of important wider environmental factors which contributed to control or conversely were found to have delayed an adequate response. Assessment of the environment or situational analyses in health planning and emergency responses are fundamental for effective design and revision of national level policies and implementation of plans based on these. The scope and content of such analyses, of course, must include basic underlying demographic, epidemiological and health metrics of the population, but also factors on the ‘supply-side’ which should account for the wider infrastructure, including technological capabilities. In the case of infectious diseases, analyses must also include the prevailing social norms and cultural context, which may pose additional risks to spread, with an understanding informing which interventions are most appropriate for breaking the chain of transmission [4]. During infectious disease outbreaks, advancements in surveillance, monitoring and modelling have enabled early warning systems and communications via the World Health Organisation (WHO), the Africa Centres for Disease Control and Prevention (Africa CDC), the European Centre for Disease Prevention and Control (ECDC), the US Centers for Disease Control and Prevention (CDC), and others. Together, they form the mechanisms for alerting the global community as outbreaks evolve to an epidemic or pandemic [5-7]. But in addition to these ‘situation reports’ (*i.e.* what is happening in terms of the disease transmission and its impact), and ideally before the emergence of a pandemic, what do we know about the capacity of a given country to respond? And how do we assess the wider contextual influences which are particularly relevant in a pandemic scenario where advanced health systems and national economies are not enough to ensure successful containment [8,9]?

Our recent work on what can be described as the ever-present pandemic threat of antimicrobial resistance, has suggested the PESTELI framework [10], which draws attention to the following environmental domains: Political factors, Economic influences, Sociological trends, Technological innovations, Ecological factors, Legislative requirements and Industry analysis [11]. These are more fully defined in **Table 1****.**

**Table 1:** Definition of PESTELI domains

Domain	Definition	Examples
Political (P)	Political commitment, political leadership, political transparency	National guidelines and policies, governance committee; accountability; corruption
Economic (Econ)	Wider economic influences which have a bearing on the health system or on individuals and organisations	Funding sources and channels
Sociological (S)	Relevant trends according to age, gender, the way people live, work, norms and behaviours. Also include factors about how professionals in organisations behave	Culture, religion, education, population composition
Technological (T)	New approaches to the surveillance, diagnosis or treatment of infections	Surveillance, diagnosis, pathogen discovery
Ecological (E)	The epidemiology of other infections and trends in human health, animal health, agricultural factors, climate	Pollution, agriculture and aquaculture, epidemiology of other diseases, vaccination
Legislative (L)	Mechanisms to support policy including the implementation of relevant legislation and effectiveness of this approach	Administrative power of health and social care organisations, travel restriction
Industry (I)	Wider industry in addition to technologies, such as pharmaceutics, investments in the health care industry, pluralistic health care (government and private share) and role of health care insurers	Workforce, medical resources, insurance, research and development (R&D)

PESTELI – Political, Economic, Sociological, Technological, Ecological, Legislative, Industry

## METHODS

We conducted a literature review to identify 1) situation analyses in pandemic management, and 2) studies which examined contextual factors influencing pandemic management. In this study, we defined ‘pandemic’ as an infectious disease outbreak that has spread across multiple continents or worldwide, affecting a large population [12,13].

### Study eligibility

Any study published in English from 01 January 2000 to 01 June 2020 that has 1) performed a situation analysis to assess the environment for pandemic management, or 2) examined macro-level contextual determinants influencing pandemic management of one or more of the following pandemics: Severe Acute Respiratory Syndrome (SARS), Middle East Respiratory Syndrome (MERS), COVID-19, Influenza A (H1N1), Ebola virus disease, and Zika virus disease, were considered in this review, in any country(ies) setting(s). The PICO (Population, Intervention, Comparison and Outcomes) [14] and SPIDER (Sample, Phenomenon of interest, Design, Evaluation, Research type) inclusion and exclusion criteria were applied at the review stages [15]. Studies focussing solely on other infectious diseases (eg*,* tuberculosis, malaria, HIV/AIDS, cholera, dengue), non-communicable conditions (eg, obesity, diabetes, Alzheimer disease, substance misuse), or local outbreaks (eg, a Methicillin-resistant *Staphylococcus aureus* outbreak in one hospital) were excluded.

### Search strategy and information sources

The methods used in this review are in line with the PRISMA extension for scoping reviews (PRISMA-ScR) guidelines [16]. The protocol is available from the authors upon request. The PRISMA-ScR checklist was completed to guide study selection and data extraction. We restricted the search period from January 2000 onwards to capture major pandemics. We limited the language to English. We searched PubMed, Ovid MEDLINE, Ovid EMBASE, Global Health, Health Management, and the Cochrane Library databases. Searches included both controlled vocabulary (pre-defined subheadings) (eg, Pandemics) and text words (eg, strategic analysis). The search strings used are provided in Appendix S1 of the **Online Supplementary Document**.

### Study selection

The title and abstract of the studies yielded from the database and reference list search were randomly assigned into two groups. Three researchers (NZ, RA, HK) participated in the title and abstract screening and in each group, by rotation, one pair independently reviewed each title and abstract and the third researcher resolved the disagreements in decisions (Group 1 - RA, Group 2 - HK). Two researchers (NZ, HK) independently reviewed the full-text articles which passed the title and abstract screening. All discrepancies were discussed and re-examined by the third reviewer (RA) until agreement was reached.

### Assessment of study quality and risks of bias

We excluded those studies where a full article was not available (eg, conference proceedings, meeting minutes). We excluded studies that did not include the sections in the preferred reporting items set out in the PRISMA-ScR checklist [16].

Formal quality appraisal of the included individual studies was not performed, as this would be beyond the aim of this scoping review, which was to map key concepts, types of evidence, and gaps in research [17,18]. Evaluation of intervention and policy effectiveness is not the aim of the current review [19,20].

### Data extraction and analysis

Three researchers (NZ, HK, RA) carried out data extraction, with cross-validation for 50% of the studies using a standardised data extraction table (Microsoft Excel, Microsoft Inc, Seattle, WA, USA). We anticipated descriptive results given the qualitative nature of the studies. Key study characteristics, methods of data collection, situational analyses frameworks employed, and which of the PESTELI domains had been examined (E), findings reported on (F) and recommendations made (R) were extracted (**Table 2**). Factors influencing pandemic management into facilitators and inhibitors against the 7 domains were synthesised (**Table 3-6**).

**Table 2:** Study design and PESTELI domains covered in individual studies

Study	Study character		Study design						PESTELI domains						
	**Year**	**Setting**	**Primary data(interviews)**	**Primary data (expert/stakeholder consensus, panel discussion)**	**Primary data (quantitative)**	**Secondary data (literature)**	**Secondary data (textual)**	**Secondary data (quantitative)**	**P**	**Econ**	**S**	**T**	**E**	**L**	**I**
**COVID-19**															
[21]	2020	Italy		*		*	*		EFR			EFR		EF	EF
[22]	2020	China					*	*				EFR			EFR
[23]	2020	USA			*						EFR				
**Ebola virus disease**															
[24]	2020	West Africa					*	*	EF	EF	EF		EFR	EFR	EF
[25]	2016	Sierra Leone	*					*	F	R	EFR	FR			FR
[26]	2014	Nigeria				*			FR		EFR	R		F	
[27]	2018	West Africa					*	*	EF	F	F				EF
**Influenza A (H1N1)**															
[28]	2018	Eastern Mediterranean		*			*		EFR	EF	F	EFR	F	EF	EF
[29]	2014	Global				*					EFR				
[30]	2010	Asia	*				*	*	EFR	EF	EF	EF	EF		EFR
[31]	2018	USA			*						EFR				
[32]	2016	Global				*					EF				
[33])	2014	Global				*					EFR				
[34]	2012	Global				*					EF				
**Multiple pandemics**															
[35]	2020	Global				*			EF	F	EFR	FR	F		EF
[36]	2020	Global				*			EF	F	FR	FR			FR
[37]	2020	Global				*	*	*	EFR		R	EFR	EFR	R	F
[38]	2012	Global				*						EFR	EFR		R
[39]	2020	Global				*			EFR	EF	EF	FR	E		

PESTILE – Political, Economic, Sociological, Technological, Ecological, Legislative, Industry, E – examined, F – findings reported, R – recommendation proposed

*Indicates types of data included in the study.

**Table 3:** Facilitators and inhibitors in pandemic management identified: COVID-19

Political (P)	Economic (Econ)	Sociological (S)	Technological (T)	Ecological (E)	Legislative (L)	Industry (I)
**COVID-19:**						
**Facilitators**						
Enactment of emergency policies and decrees (Italy) [21]			Health informatics technologies (*eg,* big data for tracking and tracing; 5G network for telemedicine; artificial intelligence for rapid, precise diagnostics); regulation of travelling using QR code of health record (China) [22]		Banned air traffic from China; mandatory reporting of travel history to the Italian National Health Service (SSN); mandatory quarantine (Italy) [21]	Rapid response including increased health care human resources capacity and protected supply chains (Italy) [21];
						High internet coverage and utilisation (China) [22]
**Inhibitors**						
Inconsistency between local and national guidance in technical orders and clinical protocols (Italy) [21]		Lack of public knowledge resulted in continuation of mass gatherings (US) [23]	Constraints in data integration and smart technologies to support contact tracing, surveillance, and other interventions (Italy) [21]			
			Lack of rapid deployment of information systems; suboptimal information exchange across heath institutions; non-standardised electronic health records to streamline emergency information (China) [22]			

**Table 4:** Facilitators and inhibitors in pandemic management identified: Ebola

Political (P)	Economic (Econ)	Sociological (S)	Technological (T)	Ecological (E)	Legislative (L)	Industry (I)
**Ebola:**						
**Facilitators**						
Political commitment contributed to a rapid/effective response in some countries (eg, Nigeria) (West Africa) [24]	Countries with trading partners are more likely to act early to protect trade and prevent contagion; securing important inputs for domestic industries or output markets motivate HCW deployment abroad (West Africa) [27]	Hand shaking discouraged by the federal government; HCWs and non-clinical staff in hospitals demanding full PPE before consulting any patient; high public awareness and interest; trust and conﬁdence in public authorities enhancing adoption of recommended containment measures (Nigeria) [26]			Temporary border closure *(eg,* Cameroon and Chad) (Nigeria) [26]	
Declaration of national emergency (eg, Nigeria); demonstration of political commitment (eg, Presidential Summit attended by Minister of Health, State Governors and their Commissioners in Nigeria); national weekly briefings to provide up-to-date information, and dispel fears, rumours and misconceptions (Nigeria) [26]						
Deployment of foreign HCWs, as aids from allies, maintain global balance of political power; historical choices and policies facilitate institutionalised capacities and norms for civil emergency management, foreign medical aid, or overseas military personnel deployments (West Africa) [27]		Media coverage and public attention facilitate humanitarian assistance and HCW deployment (West Africa) [27]				
**Inhibitors**						
Political interference (*eg,* contact tracer recruitment and organisation led by non-health institutes) (Sierra Leone) (23)	Poor health care system financing (West Africa) [24]	Inadequate self-prescribed infection preventative measures due to poor health education; poor housing conditions in rural areas; poor safety orientation (training) in hospitals; low adherence to government regulations in rural areas despite public campaigns; re-infection due to risky sexual behaviours; lack of follow-up with recovered cases and long-term monitoring; culture and tradition (eg, mass gathering at funerals) (West Africa) [24]	Incomplete case monitoring database (Sierra Leone) [25]	High prevalence of nosocomial infections; climate conditions increasing transmission; deforestation; physical proximity between human and wildlife, including animal reservoirs (eg, fruit bats); zoonotic pathogens transmitting across species; low vaccination due to misinformation in mass media (West Africa) [24]	Cross-border transmission due to relaxed immigration policies (West Africa) [24]	Inadequate drug and PPE supply; staffing limitation due to transmission among HCWs (West Africa) [24]
Contests between powerful domestic actors delaying crisis response; organisational limitations, cognitive barriers and political construction of threat perception in policy makers may lead to hesitation in HCW deployment (West Africa) [27]		Rejecting contact tracing due to stigma and fear, and/or to avoid quarantine; inadequate training of contact tracers; lack of support to quarantined citizens (Sierra Leone) [25]				Lack of appropriate equipment for contact tracers; heavy workload due to shortage of contact tracers (Sierra Leone) [25]
		Stigma and discrimination against patients and HCWs who treated them and subsequent actions (*eg,* protests near treatment centres due to lack of knowledge, fear, and misinformation on mass media (eg, Ebola infection is incurable); low willingness among HCWs to join the front line due to fear; low confidence in the capacity of health system and leadership to provide reliable information and resources for infection prevention (Nigeria) [26]				Deployment of HCWs can be delayed if industry interdependence exists, such as logistical planning, medical evacuation, and other necessities (West Africa) [27]

**Table 5:** Facilitators and inhibitors in pandemic management identified: Influenza A (H1N1)

Political (P)	Economic (Econ)	Sociological (S)	Technological (T)	Ecological (E)	Legislative (L)	Industry (I)
**Influenza A (H1N1):**						
**Facilitators**						
Arrangement and strength in governance and stewardship (Asia) [30]	External funds through the Partnership Contribution (PC) of Pandemic Influenza Preparedness (PIP) (Eastern Mediterranean) [28]	Public knowledge (*eg,* knowledge in transmission mechanism, infection control measures; efficacy and effectiveness of control measures); optimal perception of severity and vulnerability of the infection (Global) [29]	Technologies available for surveillance, case detection, and infection control (Asia) [30]	Vaccination coverage; early initiation of antivirals (Eastern Mediterranean) [28]		External resources available for LMICS (eg, Laos, Cambodia) (Asia) (31)
		Optimal knowledge in the influenza pandemic; having a health-related personal network (eg, having family or friends who can provide health-related information or support) (US) [31]		Existing epidemiological profile of high life expectancy and low mortality (Asia) [30]		
		Adherence with antiviral medication (either as prophylaxis or treatment) associated with previous compliance with other precautionary advice about pandemic flu, beliefs that the recommended preventive measures were necessary; having discussed the option of taking antiviral medication with someone who had not experienced side effects (Global) [32]				
		Perception of benefits of vaccination (eg, protecting themselves and loved ones, protecting patients); adequate perception of susceptibility (eg, risk of infection, immunity via previous exposure) and severity; responsive action to information from mass media, public health authorities, and coworkers/supervisor (Global) [34]				
**Inhibitors**						
Inadequate preparedness plans lacking detailed strategic review and assessment (Eastern Mediterranean) [28]	Insufficient budget for pandemic preparedness; reliance on external funding (Asia) [30]	The annual Islamic pilgrimage (Hajj) driving transmission; population displacement and migration due to ongoing wars and conflicts (Eastern Mediterranean) [28]	Lack of complete surveillance systems across national, sub-national and regional level; absence of integration between animal and human surveillance networks (Eastern Mediterranean) [28]	Global migratory bird flight increasing transmission of Avian influenza through wild birds, poultry and humans (Eastern Mediterranean) [28]	Absence of legal framework (for declaring emergency and taking actions) in pandemic planning (Eastern Mediterranean) [28]	Shortage in trained staff and laboratory equipment for surveillance; lack of planning for procurement, storage and distribution of vaccines; low utilisation of research and evaluation to revise preparedness plans and improve prevention and containment measures (Eastern Mediterranean) [28]
		Anxiety and fear (Global) [29]				Shortage of qualified human resources restricting surveillance and response capacity (Asia) [30]
		Lack of public health education specifically for Influenza A (instead focusing on Avian influenza) (Asia) [30]				
		Low education; unemployment and low socio-economic position associated with inadequate access to health information (US) [31]				
		Non-adherence with antiviral medication due to experienced or perceived adverse effects, not wanting to take medication, forgetting, losing, or running out of tablets (Global) [32]				
		Social stigma and discrimination against one or more particular social sub-group (s); lack of trust in government’s capacity and fairness when handling the emergence; inequalities in exposure to public health communication messages which led to negative outcomes including low vaccine uptake; inadequate knowledge, attitude, and beliefs about the pandemic; suboptimal care seeking behaviour; low ability and willingness to seek and process information; poor emotional responses (Global) [33]				
		Vaccine hesitancy among HCWs due to concerns in vaccine safety, adverse effects, effectiveness/efficacy) (Global) [34]				

**Table 6:** Facilitators and inhibitors in pandemic management identified: multiple pandemics

Political (P)	Economic (Econ)	Sociological (S)	Technological (T)	Ecological (E)	Legislative (L)	Industry (I)
**Multiple pandemics:**						
**Facilitators**						
Policies to define Community Health Worker (CHW) tasks and roles; stakeholder engagement in governance arrangements (Global) [35]		Appropriate CHW training; organised and funded well-being support to CHWs; community engagement to enhance social mobilisation, build trust and increase service utilisation; transparency in communication mitigated fears (Global) [35]	Information management systems and digital health technology employed for CHW programmes (Global) [35]	Improved vaccination coverage with as an outcome of CHWs’ regular household visits, liaising with poultry and feed sellers at marketplace (Global) [35]		Adequate PPE supply to CHWs (Global) [35]
Collaboration between governmental agencies and external organisations (eg, the CDC and WHO) (Global) [37]	Sustained investment in CHWs (eg, financial incentives remote area allowance, performance-based financing payments or accommodation); additional resources to support the well-being of CHWs during and post pandemic (Global) [35]	Community palliative care to support people who prefer to remain at home towards end of life; re-deployment of volunteers to provide psychosocial and bereavement care; support carers to deal with stress; communication and leader identification in environment with multiple caregivers, especially in low resource settings (Global) [36]	Volunteers transitioned to become virtually deployed (Global) [36]			
Credibility of evidence informing responses; health care system capacity (Global) [39]			Pathogen discovery techniques; meta-genomic technology to predict pandemic potential in novel microbes (Global) [38]			
**Inhibitors**						
Lack of a prior pandemic communication plan (Global) [35]	Ethical challenges concerning allocation of scare resources (Global) [36]	Globalisation accelerating transmission; culture (eg*,* traditional burial practices, dietary habits such as consumption of bush meat, blaming and social stigma) (Global) [39]	Non-functional surveillance systems due to delayed reporting from health facilities; contact tracing potentially hamper primary service delivery (Global) [35]	Fast transmission due to environmental change and international travel via rail and air (Global) [37]		Disruption in drug and equipment supplies common during pandemics; lack of research in equity, gender equality, and economic evaluation of CHW programmes (Global) [35]
Delayed, poor coordination of hospital level policies and protocols and hospice-specific guidance (Global) [36]	Economic inequalities in social sub-group(s) (Global) [39]		Lack of data collection systems to understand patient outcomes and share learnings (Global) [36]			Lack of material supplies (eg, PPE, diagnostic and monitoring equipment) (Global) [36]
Confusion in attribution of responsibility (eg, health care system or the general public); lack of coordination in responses among agencies due to competing causal explanations of the pandemic and conflicts in priorities (Global) [39]			Low adoption of remote medical assistance to detect and control zoonotic infectious disease outbreaks (Global) [37]	Juxtaposition of livestock production and wildlife populations; change in land use related to development and deforestation (Global) [38]		Lack of integration of internet and related technologies for surveillance activities (eg, simultaneous reporting and monitoring, end-to-end connectivity, data assortment and analysis, tracking and alerts) (Global) [37]
			Inadequate case reporting due to lack of information technologies (Global) [39]			

*[35]: Lassa, Ebola, Influenza (H1N1, H5N1); [36]: Ebola, SARS, COVID-19, Influenza (H1N1); [37]: SARS, MERS, COVID-19; [38]: HIV/AIDS, SARS, Influenza (H1N1); [39]: SARS, Zika, Ebola.

### Ethics approval

This study did not require ethics approval as is a literature review.

## RESULTS

### Included studies

**Figure 1:**
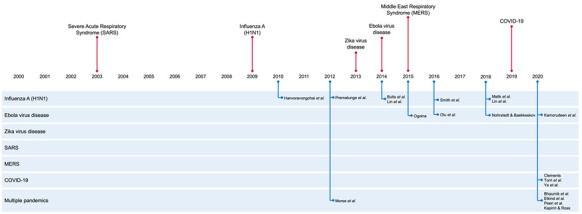
Study flowchart.

A total of 176 studies were identified from the primary electronic databases. Two further studies were identified through a search of reference lists. After removal of duplicates and studies in diseases not of interest, a total of 144 records remained for screening. 45 studies were eligible for full text review and 26 studies were excluded with reasons, yielding 19 studies that met the inclusion criteria. **Figure 1** summarizes the flow of literature search and screening.

### Study characteristics

Of the included studies, 6 were single country analyses [21-23,25,26,31], and 13 were regional level multi-country studies [24,27-30,32-39]. Fourteen studies had a single disease focus, with 3 studies on COVID-19 [21-23], 4 studies on Ebola virus disease [24-27], and 7 studies on Influenza A [28-34]. Five studies evaluated responses to one or more of COVID-19, SARS, MERS, Influenza A (H1N1), Ebola virus disease, and Zika virus disease pandemics [35-39].

**Figure 2:**
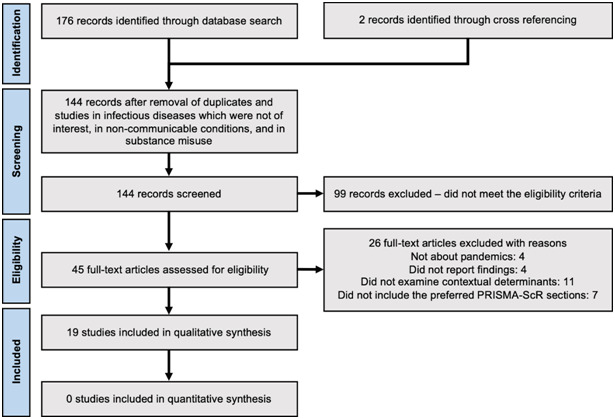
Pandemic and study publication timeline.

No study included in this review explicitly set out to employ the PESTELI framework, but 3 studies employed alternative frameworks, including the SWOT (Strengths, Weaknesses, Opportunities, and Threat) framework [21], the PIP (Pandemic Influenza Preparedness) framework [28], and the SYSRA (Systemic Rapid Assessment) framework [30]. The other 16 studies examined macro-level determinants affecting the response and ability to manage the pandemic, including workforce mobilisation and deployment; adherence of vaccination and antiviral therapy; public knowledge, awareness, and perception; and compliance of non-pharmaceutical interventions. All studies were published after pandemic emergence. The timeline of the pandemics against the publication of the included studies (**Figure 2**), shows a notable gap for SARS and Zika.

Most of the studies employed one method of data collection: 9 reviews of the published academic literature [26,32-36,38-40] and 2 [23,31] used primary data through population survey surveys.

Four studies [21,25,28,30] used primary data via interviews or panel discussion with experts and stakeholders as well as secondary data collected through review of literature or other textual sources.

Ten studies were results of work by researchers from a single country [21-23,26,29,32-34,36,39]. Nine studies were outcomes of international collaborations, where all corresponding authors of these international study groups were from high income countries bar 1 [24,25,27,28,30,31,35,37,38]. Two studies involved co-operation between research institutes and international agencies (ie, WHO and UN) [25,28]. Two studies had co-authors from national and local health authorities [21,25]. One study bridged research institutes, national and local health authorities, and the private sector [37].

### Analysis using the PESTELI framework

Though the PESTELI framework was not utilised, one study reported findings in each of the domains [28]. Most studies (16) included analysis of the sociological domain. Notable gaps are evident in the legislative (14 studies), ecological (12 studies) and economic (11 studies) domain. While the political domain features in 11 studies [21,24-28,30,35-37,39] only five of these make recommendations in this domain.

Political facilitators influencing the response included demonstration of political commitment [21,24,26], and strength in governance and stewardship [30]. Inhibitors within the political domain emanated from lack of coordination between central and local governments and inadequate preparedness plans (21,36); discord about which experts and institutes should lead [25] and the extent of inclusivity of stakeholders [35].

Under economic factors, international aid and external funds were a facilitator (28,29) but over reliance on external funding was also reported as a barrier [30]. Level of health system financing was an inhibitor [24,30] and facilitator depending on country context, particularly in regards to sustained community health worker investment and enhanced support during pandemics in the case of Ebola in Uganda and Sierra Leone [35].

Sociological facilitators were high media coverage and maintaining public attention [27]; professional training of staff in health care and social care organisations [35]; and social support to citizens in isolation [36]. Conversely, the most frequently reported sociological inhibitors include lack of public knowledge and public health education in infectious disease prevention [23,26,30,31]; stigma and discrimination against infected patients and health care professionals involved in direct patient care [25,26,33,39]; cultural, traditional, and/or religious practices that may over-ride guidance and health protection messages [24,28,39]. Perceived low risk of infection threat and the low value of infection preventive measures [32,34], and, diametrically opposite, anxiety and fear [26,29], also hindered progress. Lack of trust and confidence in authorities and abilities of the health care system to cope affected health-seeking behaviours [26,33]. Recommendations were proposed in 9 studies to address these sociological inhibitors, and some repeated from the first of these studies in 2014 to the latest in 2020. Recommendations include transparent communication between government and citizens to share information that is up-to-date, easy to interpret, and relevant to contexts (*eg,* tailored information for vulnerable groups) [23,25,26,29,31,33,35-37].

Among the 7 studies, which included ecological analysis, 6 also analysed sociological factors [24,28,30,35,37,39]. The findings suggested that the drastic change in human lifestyle exerted an impact on ecological and environmental profiles, which then influenced human behaviour further. For instance, globalisation (S) and deforestation and climate change (E); dietary habits (S) and livestock production (E); population age distribution (S) and epidemiology profile (E); and international travel (S) and infection transmission (E). High vaccination coverage was the only ecological facilitator reported in 3 studies [28,30,35]. Ecological inhibitors were centred around human behaviour; contact/proximity with wild animals; transmission of zoonotic diseases through livestock production, and high levels of international travel [24,28,37,38].

Among the 11 studies which assessed factors in the technological domain [21,22,25,26,28,30,35-39], existing information technologies did facilitate progress [22,30], but delayed deployment and limited utilisation of such technologies remained an inhibitor resulting in weak surveillance capacity [21,22,25,28,35,36,39]. In terms of the wider industry, internet coverage was cited as a facilitator [22] and inhibitor when coverage was low [37]. Industry inhibitors were an inadequate supply of personal protective equipment (PPE) and other medical resources [24,25,27,35,36]; and medical staff shortages [24,25,28,30]. As expected, the interdependence between the technological and industry domains is highlighted. Technologies reliant on uninterrupted power and network coverage are obvious examples, but also more basic equipment and supply-and-distribution chains rely on the existing wider industry or the ability to quickly scale up and deploy emergency provisions. Recommendations, including, for example, accelerated mobilisation of research and development (R&D) through incentives, were proposed to mitigate inhibitors in both technological and industry domains to enhance preparedness for future pandemics [22], but the timescales for this varied.

Overall, as noted above, the legislative domain was a gap in analyses and also was not explicitly assessed in the otherwise comprehensive assessment using the SYSRA framework of the Influenza A pandemic [30]. Five studies reported legislative facilitators [21,24,26,28,37] including travel bans and border closures [21,24,26]. The absence of legal frameworks for declaring an emergency and taking actions was cited as an inhibitor in the Eastern Mediterranean region [28].

## DISCUSSION

Our findings appear to show missed opportunities for capture and synthesis of learning, based on a comprehensive analysis within and across pandemics. Wider and more timely dissemination of learning is needed. Large time delays between pandemic event and analysis are evident (see **Figure 2**). There are recommendations that had been made, from the relatively sparse set of studies, but which now appear again in the current pandemic as inhibitors across the 7 domains. This slow knowledge mobilisation has contributed to the apparent lack of preparedness in many countries for the current COVID-19 pandemic [41,42]. The vast range of outputs chosen for situational analyses could be interpreted as a signal that the endeavour is somehow seen as less scientific, or that the application of strategic management analyses in health has yet to mature. Public health journals have provided rapid turnaround on numerous opinion pieces which may have contributed to a disparate body of work lacking a common framework for synthesis. Additionally, this vacuum has left social media platforms as a fertile ground for debate on these macro-level influences [43]. We encourage a more robust and comparable approach. Additionally, data sources used for analyses are largely confined to secondary sources with only 6 studies employing primary and secondary or mixed methods approaches, which means that findings do not benefit from multi-disciplinary inquiry and the necessary data triangulation. While the PESTELI framework is designed to help draw out the influences specific to each domain, the approach also highlights the interconnections and complexity between the domains. The idea of interconnectivity is certainly not a new one when looking at health systems strengthening [2,44]. For example, inclusion of wider industry experts including project managers, data analysts, engineers, and experts in health systems and applied system methodologies must be coupled with the advocacy work and mobilisation of ‘thought leaders’ [2]. We have recently been urged to use this crisis as an opportunity to equip and strengthen the system. The role of social care in this wider definition of health systems needs to be made more explicit. This review unveiled the missed opportunity in integrating community-based care and collaborating with social care organisations in the previous Ebola pandemic and in high income countries in particular, in the current COVID-19 pandemic. The sector was not only underprepared but also inadequately supported, a concern raised well before the COVID-19 pandemic [45].

We acknowledge that limiting the study language has missed some national/local level studies but made this decision as the aim here was to look at potential for international learning. We encourage future analysis to include studies published in different languages and assess how the facilitators and inhibitors across the PESTELI domains might influence pandemic responses differently in world regions.

While this review was confined to the lessons from emergent pandemics since 2000, previous pandemics, notably HIV, provide us with key lessons about the importance of protecting the most vulnerable groups and the impressive economic gains when a global health coordinated perspective is taken. We need to capture the lessons which enabled that novel threat to be not only contained but also integrated in the planning of robust, holistic health and social care provision, with the political, sociological and technological domains working over time. Further within- and cross-domain analysis may be strengthened using established assessment tools, for example, the governance TAPIC (Transparency, Accountability, Participation, Integrity, Capacity) framework [46], building on previous work and enhancing comparability. The traditional use of such analysis in management sciences is then to guide a force-field analysis where strategies are formulated to either weaken the inhibitors or strengthen the facilitators whilst also explicitly acknowledging which factors are immutable for the short or medium term. Where political or economic barriers are unlikely to change (as evident by the lack of recommendations in these domains), these constraints are still useful when projecting potential impacts of the programmes with a sociological or technological focus, for example. As we learn and adjust to this novel pandemic we need to prepare for the short, medium and long-term and the framework suggested here can help with the required 360-degree view.

## CONCLUSIONS

Ex-post analysis using the seven-domain strategic management framework provides further opportunities for a planned systematic response to pandemics which remains critical as the current COVID-19 pandemic evolves.

## Additional material

Online Supplementary Document
