# Supplementary material for: Macro level factors influencing strategic responses to emergent pandemics: A scoping review
Source: J Glob Health. 2021 Jul 1;11:05012. doi: 10.7189/jogh.11.05012 (PMC8248748; doi:10.7189/jogh.11.05012)
Supplement: Online Supplementary Document [file jogh-11-05012-s001.pdf]

Online Supplementary Document

Table S1. Scoping review search strings

Strings for PubMed:

Search 1:

((situation analysis[Title/Abstract]) OR (situational analysis[Title/Abstract]) OR (strategic analysis[Title/Abstract]) OR (SWOT[Title/Abstract]) OR (PESTEL[Title/Abstract]) OR (PESTELI[Title/Abstract]) OR (STEEPLE[Title/Abstract])) OR (PEARLES[Title/Abstract])
AND
((pandemic*[Title]) OR (epidemic*[Title]) OR (outbreak*[Title]))

Search 2:

((preparedness*[Title]) OR (planning[Title]) OR (response*[Title]) OR (control[Title]) OR (mitigate*[Title]) OR manag*[Title])
AND
((facilitat* [Title/Abstract]) OR (inhibit*[Title/Abstract]) OR (influenc*[Title/Abstract]) OR (correlate*[Title/Abstract]) OR (determinant*[Title/Abstract]) OR (predictor*[Title/Abstract]) OR (barrier*[Title/Abstract]) OR (contribut*[Title/Abstract]) OR (driv*[Title/Abstract]))
AND
((anthropolog*[Title/Abstract]) OR (socio*[Title/Abstract]) OR (social*[Title/Abstract]) OR (politic*[Title/Abstract]) OR (economic*[Title/Abstract]) OR (technolog*[Title/Abstract]) OR (ecolog*[Title/Abstract]) OR (environment*[Title/Abstract]) OR (legislati*[Title/Abstract]) OR (law*[Title/Abstract]) OR (regulat*[Title/Abstract]) OR (industr*[Title/Abstract]))
AND
((pandemic*[Title]) OR (epidemic*[Title]) OR (outbreak*[Title]))
Limit: 2000-current
Limit: English only

Strings for Ovid databases:

Search 1:

1. (situation analysis or situational analysis or strategic analysis or SWOT or PESTEL or PESTELI or STEEPLE or PEARLES).ab. and (pandemic* or epidemic* or outbreak*).ti.
2. limit 1 to English language
3. limit 2 to yr = "2000-Current"

Search 2:

1. (((prepare* or planning or respon* or control* or mitigate* or manag*) and (facilitat* or enabl* or inhibit* or influenc* or correlate* or determinant* or predictor* or barrier* or contribut* or driv*) and (anthropolog* or

```
socio* or social* or politic* or economic* or technolog* or ecolog* or environment* or legislati* or law* or
regulat* or industr*)).ab. and (pandemic* or epidemic*).ti.) not (diabet* or asthma or opioid* or obes* or HIV or
malaria or tuberculosis).ti.
2. limit 1 to english language
3. limit 2 to yr = "2000-Current"
```

Table S2. Facilitators and inhibitors in pandemic management identified in individual studies

(a) COVID-19

	Political (P)	Economic (Econ)	Sociological (S)	Technological (T)	Ecological (E)	Legislative (L)	Industry (I)
**COVID-19**							
(21)	**Facilitators**						
	Enactment of emergency policies and decrees					Banned air traffic from China; mandatory reporting of travel history to the Italian National Health Service (NHS); mandatory quarantine	Rapid response including increased healthcare human resources capacity and protected supply chains
	**Inhibitors**						
	Inconsistency between local and national guidance in technical orders and clinical protocols			Constraints in data integration and smart technologies to support contact tracing, surveillance, and other interventions			
(22)	**Facilitators**						
				Health informatics technologies (*e.g.* big data for tracking and tracing; 5G network for telemedicine; artificial intelligence for rapid, precise diagnostics); regulation of travelling using QR code of health record			High internet coverage and utilisation
	**Inhibitors**						
				Lack of rapid deployment of information systems; suboptimal information exchange across heath institutions; non-standardised electronic health records to streamline emergency information			
(25)	**Facilitators**						
	**Inhibitors**						

			Lack of public knowledge resulted in continuation of mass gatherings				

(b) Ebola

	Political (P)	Economic (Econ)	Sociological (S)	Technological (T)	Ecological (E)	Legislative (L)	Industry (I)
**Ebola**							
	**Facilitators**						
	Political commitment contributed to a rapid/effective response in some countries (*e.g.* Nigeria)						
	**Inhibitors**						
(27)		Poor healthcare system financing	Inadequate self-prescribed infection preventative measures due to poor health education; poor housing conditions in rural areas; poor safety orientation (training) in hospitals; low adherence to government regulations in rural areas despite public campaigns; re-infection due to risky sexual behaviours; lack of follow-up with recovered cases and long-term monitoring; culture and tradition (*e.g.* mass gathering at funerals)		High prevalence of nosocomial infections; climate conditions increasing transmission; deforestation; physical proximity between human and wildlife, including animal reservoirs (*e.g.* fruit bats); zoonotic pathogens transmitting across species; low vaccination due to misinformation on mass media	Cross-border transmission due to relaxed immigration policies	Inadequate drug and PPE supply; staffing limitation due to transmission among HCWs
	**Facilitators**						
	**Inhibitors**						
(23)	Political interference (*e.g.* contact tracer recruitment and organisation led by non-health institutes)		Rejecting contact tracing due to stigma and fear, and/or to avoid quarantine; inadequate training of contact tracers; lack of support to quarantined citizens	Incomplete case monitoring database			Lack of appropriate equipment for contact tracers; heavy workload due to shortage of contact tracers
(24)	**Facilitators**						

	Declaration of national emergency (*e.g.* Nigeria); demonstration of political commitment (*e.g.* Presidential Summit attended by Minister of Health, State Governors and their Commissioners in Nigeria); national weekly briefings to provide up-to-date information, and dispel fears, rumours and misconceptions		Hand shaking discouraged by the federal government; HCWs and non-clinical staff in hospitals demanding full PPE before consulting any patient; high public awareness and interest; trust and confidence in public authorities enhancing adoption of recommended containment measures			Temporary boarder closure (*e.g.* Cameroon and Chad)	
	**Inhibitors**						
			Stigma and discrimination against patients and HCWs who treated them and subsequent actions (*e.g.* protests near treatment centres due to lack of knowledge, fear, and misinformation on mass media (*e.g.* Ebola infection is incurable); low willingness among HCWs to join the front line due to fear; low confidence in the capacity of health system and leadership to provide reliable information and resources for infection prevention				
(28)	**Facilitators**						
	Deployment of foreign HCWs, as aids from allies, maintain global balance of political power; historical choices and policies facilitate institutionalised capacities and norms for civil emergency management, foreign medical aid, or overseas military personnel deployments	Countries with trading partners are more likely to act early to protect trade and prevent contagion; securing important inputs for domestic industries or output markets motivate HCW deployment abroad	Media coverage and public attention facilitate humanitarian assistance and HCW deployment				
	**Inhibitors**						

	Contests between powerful domestic actors delaying crisis response; organisational limitations, cognitive barriers and political construction of threat perception in policy makers may lead to hesitation in HCW deployment						Deployment of HCWs can be delayed if industry interdependence exists, such as logistical planning, medical evacuation, and other necessities

(c) Influenza A (H1N1)

	Political (P)	Economic (Econ)	Sociological (S)	Technological (T)	Ecological (E)	Legislative (L)	Industry (I)
				**Influenza A (H1N1)**			
(32)	**Facilitators**						
	External funds through the Partnership Contribution (PC) of pandemic influenza preparedness (PIP)			Vaccination coverage; early initiation of antivirals			
	**Inhibitors**						
	Inadequate preparedness plans lacking detailed strategic review and assessment		The annual Islamic pilgrimage (Hajj) driving transmission; population displacement and migration due to ongoing wars and conflicts	Lack of complete surveillance systems across national, sub-national and regional level; absence of integration between animal and human surveillance networks	Global migratory bird flight increasing transmission of Avian influenza through wild birds, poultry and humans	Absence of legal framework (for declaring emergency and taking actions) in pandemic planning	Shortage in trained staff and laboratory equipment for surveillance; lack of planning for procurement, storage and distribution of vaccines; low utilisation of research and evaluation to revise preparedness plans and improve prevention and containment measures
(33)	**Facilitators**						
			Public knowledge (*e.g.* knowledge in transmission mechanism, how to infection control measures; efficacy and effectiveness of control measures); optimal perception of severity and vulnerability of the infection				
	**Inhibitors**						

			Anxiety and fear				
(34)	**Facilitators**						
	Arrangement and strength in governance and stewardship			Technologies available for surveillance, case detection, and infection control	Existing epidemiological profile of high life expectancy and low mortality		External resources available for LMICS (*e.g.* Laos, Cambodia)
	**Inhibitors**						
		Insufficient budget for pandemic preparedness; reliance on external funding	Lack of public health education specifically for Influenza A (instead focusing on Avian influenza)				Shortage of qualified human resources restricting surveillance and response capacity
(26)	**Facilitators**						
			Optimal knowledge in the influenza pandemic; having a health-related personal network (*e.g.* having family or friends who can provide health-related information or support)				
	**Inhibitors**						
			Low education; unemployment and low socio-economic position associated with inadequate access to health information				
(35)	**Facilitators**						
			Adherence with antiviral medication (either as prophylaxis or treatment) associated with previous compliance with other precautionary advice about pandemic flu, beliefs that the recommended preventive measures were necessary; having discussed the option of taking antiviral medication with someone who had not experienced side effects				

	**Inhibitors**						
			Non-adherence with antiviral medication due to experienced or perceived adverse effects, not wanting to take medication, forgetting, losing, or running out of tablets				
(36)	**Facilitators**						
	**Inhibitors**						
			Social stigma and discrimination against one or more particular social sub-group (s); lack of trust in government's capacity and fairness when handling the emergence; inequalities in exposure to public health communication messages, which led to negative outcomes, including low vaccine uptake; inadequate knowledge, attitude, and beliefs about the pandemic, suboptimal care seeking behaviour; low ability and willingness to seek and process information; poor emotional responses				
(37)	**Facilitators**						
			Perception of benefits of vaccination (*e.g.* protecting themselves and loved ones, protecting patients); adequate perception of susceptibility (*e.g.* risk of infection, immunity via previous exposure) and severity; responsive action to information from mass media, public health authorities, and co-workers/supervisor				
	**Inhibitors**						

			Vaccine hesitancy among HCWs due to concerns in vaccine safety, adverse effects, effectiveness/efficacy)				

(d) Multiple pandemics

	Political (P)	Economic (Econ)	Sociological (S)	Technological (T)	Ecological (E)	Legislative (L)	Industry (I)
				**Multiple pandemics**			
(38)	**Facilitators**						
	Policies to define CHW tasks and roles; stakeholder engagement in governance arrangements	Sustained investment in CHWs (*e.g.* financial incentives remote area allowance, performance-based financing payments or accommodation); additional resources to support the wellbeing of CHWs during and post pandemic	Appropriate CHW training; organised and funded wellbeing support to CHWs; community engagement to enhance social mobilisation, build trust and increase service utilisation; transparency in communication mitigated fears	Information management systems and digital health technology employed for CHW programmes	Improved vaccination coverage with as an outcome of CHWs' regular household visits, liaising with poultry and feed sellers at marketplace		Adequate PPE supply to CHWs
	**Inhibitors**						
	Lack of a priori pandemic communication plan			Non-functional surveillance systems due to delayed reporting from health facilities; contact tracing potentially hamper primary service delivery			Disruption in drug and equipment supplies common during pandemics; lack of research in equity, gender equality, and economic evaluation of CHW programmes
(39)	**Facilitators**						
			Community palliative care to support people who prefer to remain at home towards end of life; re-deployment of volunteers to provide psychosocial and bereavement care; support carers to deal with stress	Volunteers transitioned to become virtually deployed			
	**Inhibitors**						

	Delayed, poor coordination of hospital level policies and protocols and hospice-specific guidance	Ethical challenges concerning allocation of scare resources		Lack of data collection systems to understand patient outcomes and share learnings			Lack of material supplies (*e.g.* PPE, diagnostic and monitoring equipment)
(29)	**Facilitators**						
	Collaboration between governmental agencies and external organisations (*e.g.* the CDC and WHO)						
	**Inhibitors**						
				Low adoption of remote medical assistance to detect and control zoonotic infectious disease outbreaks	Fast transmission due to environmental change and international travel via railway and air way		Lack of integration of internet and related technologies for surveillance activities (*e.g.* simultaneous reporting and monitoring, end-to-end connectivity, data assortment and analysis, tracking and alerts)
(30)	**Facilitators**						
				Pathogen discovery techniques; meta-genomic technology to predict pandemic potential in novel microbes			
	**Inhibitors**						
					Juxtaposition of livestock production and wildlife populations; change in land use related to development of tropical forests		
(31)	**Facilitators**						
	Credibility of evidence informing responses; healthcare system capacity						
	**Inhibitors**						

	Confusion in attribution of responsibility (*e.g.* healthcare system or the general public?); lack of coordination in responses among agencies due to competing causal explanations of the pandemic and conflicts in priorities	Economic inequalities in social sub-group(s)	Globalisation accelerating transmission; culture (*e.g.* traditional burial practices, dietary habits such as consumption of bush meat, blaming and social stigma)	Inadequate case reporting due to lack of information technologies			
